# A one health perspective on multidrug-resistant bacterial infections: integrated approaches for surveillance, policy and innovation

**DOI:** 10.3389/fcimb.2025.1614232

**Published:** 2025-09-03

**Authors:** Chinenyenwa M. D. Ohia, Olutayo Israel Falodun, Lucky Icomiare Adebudo, Adeleye Solomon Bakarey

**Affiliations:** ^1^ Department of Environmental Health Sciences, Faculty of Public Health, College of Medicine, University of Ibadan, Ibadan, Oyo, Nigeria; ^2^ Nanobios Lab, Department of Biosciences and Bioengineering, Indian Institute of Technology (IIT), Bombay, India; ^3^ Department of Microbiology, University of Ibadan, Ibadan, Nigeria; ^4^ Edo State Ministry of Agriculture and Food Security, Benin, Edo, Nigeria; ^5^ Edo State Ministry of Agriculture and Food Security Animal Ethics Committee, Benin, Nigeria; ^6^ Institute for Advanced Medical Research and Training (IAMRAT), College of Medicine, University of Ibadan, Ibadan, Nigeria

**Keywords:** multidrug-resistant (MDR) bacteria, one health, antimicrobial stewardship, environmental reservoirs, AI technologies, inter-sectoral collaborations, zoonotic MDR outbreaks, global health

## Abstract

Multidrug-resistant (MDR) bacterial infections represent a growing global health emergency, driven by interconnected human, animal, and environmental factors. This review adopts a One Health perspective to explore the transmission dynamics, operational integration, and innovative responses to antimicrobial resistance (AMR). Case studies from China, India, Nigeria, Thailand, and Brazil underscore the effectiveness of regulatory reforms, surveillance networks, and public engagement campaigns. Notably, Artificial Intelligence (AI) is transforming MDR management through real-time diagnostics and resistance prediction, though ethical concerns and infrastructure deficits in Low- and Middle-Income Countries (LMICs) remain barriers. Community-led initiatives, gender-sensitive education, and policy reforms are vital to curbing misuse and closing equity gaps. Despite successes, challenges such as fragmented governance, underfunded labs, and limited longitudinal research persist. A proactive, integrated One Health approach—linking clinical, environmental, and policy actions—is essential for reducing MDR burden. Investment in intersectoral surveillance, equitable AI deployment, and community empowerment is imperative for safeguarding antibiotics and ensuring global health resilience.

## Introduction

The increasing threat of Multidrug-resistant (MDR) bacterial infections poses a global health emergency, with about 4.95 million deaths recorded annually linked to antimicrobial resistance (AMR) (Murray et al., 2022). This is driven by the interconnected dynamics of human, animal, and environmental health factors (Berendonk et al., 2015; WHO, 2023; Nigeria Centre for Disease Control, 2024). It is projected that without strategic interventions, deaths due to AMR infections could rise to 10 million per year with up to $100 trillion in economic losses by 2050 (O'Neill, 2016; Murray et al., 2022).

Clinically, the misuse and over-prescription of antibiotics, alongside rising hospital-acquired infections, drive MDR proliferation, leading to increased healthcare costs and poorer patient outcomes. While Antimicrobial Stewardship Programs (ASPs) offer some mitigation, they remain insufficient without broader systemic integration. In the environment, resistant bacteria persist and spread through agricultural runoff, industrial waste, and untreated sewage. This emphasizes the urgent need for enhanced waste management and stricter controls on antibiotic use in agriculture.

On a global scale, MDR transmission is exacerbated by international travel, trade, and healthcare inequities, particularly in LMICs (Patel et al., 2022; Yopa et al., 2023). Although global initiatives by WHO, FAO, and OIE have laid a foundation for action, significant gaps in policy, surveillance, and cross-sector collaboration remain. Hence, addressing this complex crisis requires a holistic approach that encompasses and integrates the drivers of MDR.

The aim of this paper is to describe the different drivers of MDR, explore the critical need for an integrated One Health approach and proffer solutions in combating MDR bacterial infections across LMICs.

## Methodology

**Figure 1:**
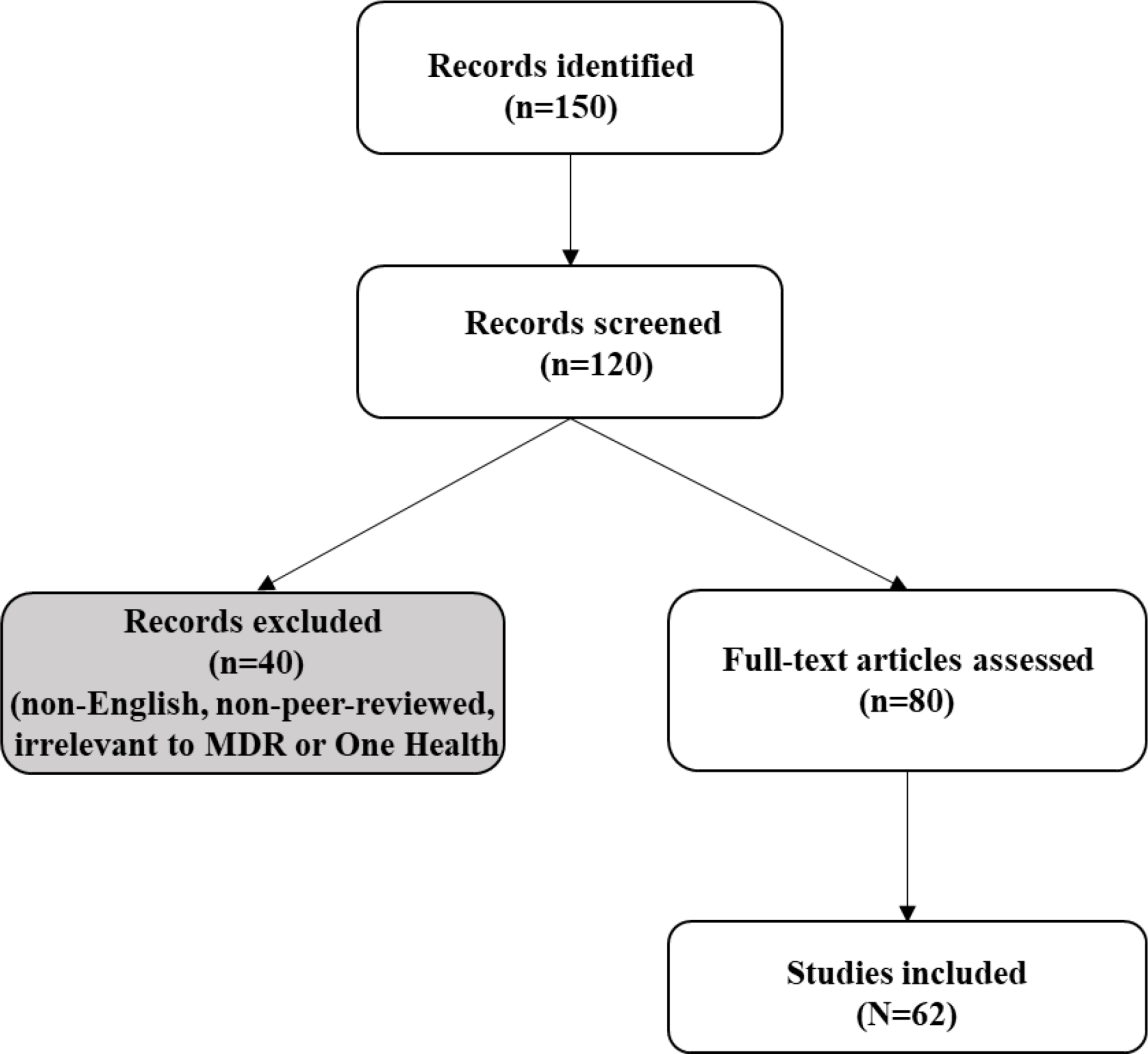
Stages of the literature selection process.

A preliminary search of literature was carried out to determine the need for the study. This was used to refine the topic within the broader concept of MDR infections. The literature searches were conducted using PubMed, Scopus, and Google Scholar (2015–2025) using keywords: “Multidrug-resistant bacteria”, “One Health”, “antimicrobial resistance”, “environmental AMR”, “AI in AMR” (**Table 1**). Inclusion criteria targeted English-language studies on MDR drivers, interventions, or case studies; non-peer-reviewed sources were excluded. Two authors independently screened 150 articles, resolving discrepancies via consensus, with 62 included after full-text review (**Figure 1**). Thematic synthesis categorized data into clinical, environmental, global, and One Health perspectives, prioritizing case studies and surveillance data. Independent screening minimized bias, ensuring a reproducible synthesis. Furthermore, the snowballing technique was used to retrieve other relevant published articles from references obtained from the databases.

**Table 1:** Literature search strategy.

Component	Details
Databases	PubMed, Scopus, Google Scholar
Keywords	“Multidrug-resistant bacteria”, “One Health”, “antimicrobial resistance”, “environmental AMR”, “AI in AMR”
Time Frame	2015–2025
Inclusion	English, peer-reviewed, MDR-focused
Coding Categories	Clinical misuse, environmental reservoirs, global spread, One Health interventions

## Discussion

### Clinical perspective

#### Antibiotic misuse and hospital-acquired infections

The prevalence of MDR bacterial infections is majorly influenced by human healthcare practices, societal behaviors, and veterinary contributions. Central to this crisis is the overuse and misuse of antibiotics in both clinical and agricultural settings. In human healthcare, antibiotics are often prescribed inappropriately, such as for viral infections where they have no efficacy, leading to the proliferation of resistant bacterial strains (Patel et al., 2022; Ventola, 2015). In addition, the prevalence of Hospital-Acquired Infections (HAIs) in long-term healthcare facilities (Yopa et al., 2023), with MDR pathogens like *Klebsiella pneumoniae*, *Acinetobacter baumannii*, and *Pseudomonas aeruginosa* demonstrating resistance to last-line antibiotics such as carbapenems (Mbimenyuy et al., 2023; Nigeria Centre for Disease Control, 2024). Resistance to other classes of antibiotics, including polymyxins and aminoglycosides, has further limited treatment options, leaving clinicians with fewer effective therapies (Sekar and Mythreyee, 2012; Tran et al., 2023). Compounding this issue are patient expectations, inadequate and sometimes lack of rapid diagnostic tools to aid in the proper use of antibiotics increasing acceptance of telemedicine by both healthcare providers and patients and systemic inefficiencies that pressure healthcare providers into unnecessary antibiotic prescriptions (WHO, 2017a; Sekar and Mythreyee, 2012; WHO, 2017b; World Health Organization (WHO), 2019).

#### Limitations of antimicrobial stewardship programs

Antimicrobial stewardship (ASP) refers to interventions designed to promote the optimal use of antibiotic agents, including drug choice, dosing, route, and duration of administration. ASPs are critical initiatives designed to optimize the use of antibiotics within healthcare settings, with the overarching goal of curbing the rise of AMR. Although, these have been documented to reduce resistance by 11–38% in high-income countries, there remain significant hurdles in LMICs (Baur et al., 2017). In sub-Saharan Africa, only 30% of countries have functional ASPs, constrained by diagnostic shortages and counterfeit drugs, which comprise 32% of antibiotics in Cameroon (Basco, 2004; Laxminarayan et al., 2013). In a rural Kenyan hospital, ASP implementation failed when clinicians, lacking resistance testing, reverted to outdated protocols, leading to treatment failures (Shamas et al., 2023). Weak enforcement, limited clinician training, and resource constraints underscore the need for a broader coordination framework to bolster stewardship efforts.

#### Veterinary contributions to MDR infections

The role of veterinary practices in the MDR crisis is a critical but often underappreciated component of the broader clinical perspective. Antibiotics are widely used in animal husbandry, aquaculture, and veterinary medicine, not only for treating infections but also as a routine measure to enhance productivity. Veterinary antibiotic use, particularly as growth promoters, fosters resistant *Escherichia coli*, transferring to humans via food chains or direct contact (Van Boeckel et al., 2015). In Nigeria, livestock antibiotic use surged 40% from 2010–2012, driven by lax regulations, with farmers purchasing over-the-counter drugs without veterinary oversight (Adesokan et al., 2015). Manure runoff contaminates water sources, creating a critical One Health interface where veterinary practices amplify environmental resistance (Woolhouse et al., 2015).

#### Resistance mechanisms

Bacteria employ different mechanisms in achieving resistance (**Table 2**). These include enzymatic degradation (e.g., beta-lactamases), target site alterations, efflux pumps, biofilms, and horizontal gene transfer, rendering treatments ineffective (Zhang and Cheng, 2022). Bacteria prevent antibiotic accumulation in their cells through limiting the entrance of drugs into bacterial cells. The porin channels in the outer membrane of the Gram-negative bacteria enables them to allow certain antibiotics such as like B-lactams and quinolones to penetrate the bacterial cells. Reduction in the number of bacterial porins could serve as means of hindrance against the antibiotics entering the cell thereby increasing the rate of resistance to the drugs (Darby et al., 2023). In addition, when the bacteria develop resistance to antibiotics through the modification of the ribosomal 30S or 50S subunits which in turn affects the production of protein. Some of the antibiotics that are resisted by this mechanism include: aminoglycosides, tetracycline, macrolides, chloramphenicol, lincosamides, and streptogramin (Sodhi and Singh, 2022; Sodhi et al., 2023).

**Table 2:** Mechanisms of antibiotic resistance.

Mechanism	Description	Example pathogens
Enzymatic Degradation	Enzymes inactivate antibiotics	*E. coli* (beta-lactamases)
Target Site Alteration	Modified binding sites reduce efficacy	*S. aureus* (MRSA)
Efflux Pumps	Expel antibiotics from cells	*P. aeruginosa*
Biofilm Formation	Protective matrices shield bacteria	*K. pneumoniae*
Horizontal Gene Transfer	Resistance genes shared across species	*Enterobacteriaceae* (mcr-1)
Modifications to the ribosomal 30s or 50s subunits	Affects production of protein	*Staphylococcus aureus*
Prevention of antibiotic accumulation in cells	Limiting the entrance of drugs into bacterial cells	Gram-negative bacteria

*Adapted from (Zhang and Cheng, 2022).

These mechanisms of resistance driven by clinical and veterinary misuse, require integrated solutions. It is important to note that although clinical practices drive MDR, environmental reservoirs amplify its spread, necessitating an exploration of ecological pathways.

### Environmental perspective

#### Pathways and hotspots

The environment functions both as a reservoir and a conduit for resistant bacteria and resistance genes. It provides a complex interface where microbial evolution, anthropogenic pollution, and public health intersect (**Table 3**). However, the environment is often overlooked with very deleterious repercussions to public health. An exploration of the environmental dimensions of MDR bacterial infections emphasizes the pathways of the introduction of resistance, its maintenance and transmission throughout the environment. Within an integrated framework, it is very important to highlight environmental hotspots of AMR, the associated risks to human and animal health and their implications for surveillance and policy development.

**Table 3:** Environmental AMR drivers and consequences.

Driver	Source	Public health consequence	Reference
Effluent Discharge	Pharmaceutical plants	*blaNDM-1* in water bodies	(Silva et al., 2024)
Agricultural Runoff	Manure, irrigation	Foodborne MDR infections	(Ehsan, 2025)
Untreated Sewage	Urban waste	Drinking water contamination	(Bjarnsholt et al., 2022)
Untreated waste water	Urban water system	Contamination of urban rivers	(Kumar et al., 2020)

*Data from environmental studies.

Wastewater treatment plants, agricultural soils, and aquatic ecosystems serve as MDR reservoirs, perpetuating resistance cycles (Wang et al., 2022). In Brazil, pharmaceutical effluents contaminate rivers with *blaNDM-1*, while untreated irrigation water in the Middle East spreads resistant bacteria to crops (Alaali and Thani, 2020; Silva et al., 2024). Agricultural runoff, laden with antibiotics from manure, fosters resistance genes, impacting food and water safety across ecosystems (Ehsan, 2025). The aquatic ecosystem (rivers, lakes, ponds etc.) serves as reservoir of resistant pathogens with very high potential as re-entry points of these pathogens into human populations through irrigation, domestic water supply for drinking, recreational activities and food production lines. In addition, contaminated drinking water, crops, and aerosols expose humans to these MDR bacteria, particularly in LMICs with poor sanitation (Bjarnsholt et al., 2022). In India, 60% of urban water sources harbor resistant bacteria, directly linking environmental contamination to clinical infections (Kumar et al., 2020; Hamer and Coffin, 2021).

Mitigation strategies such as surveillance of resistance genes, stricter waste discharge regulations, and composting practices can reduce environmental AMR (Tsekleves et al., 2023). Public education campaigns on exposure risks, such as avoiding contaminated water sources, further strengthen prevention efforts (WHO, 2023).

### Global health perspective

#### Global spread via travel and migration

Environmental spread of MDR bacterial infection is compounded by global dynamics, where travel (both human and animal migration) and inequities accelerate MDR’s reach. For instance, in 2018, 1.4 billion tourists facilitated the global spread of *NDM-1* and *mcr-1*, with Saudi Arabia’s medical tourism linked to hospital-acquired infections (Payne et al., 2016; Arcilla et al., 2017). Cross-border patient transfers, such as between the Netherlands and Germany, face hygiene discrepancies, complicating infection control (Müller et al., 2015).

#### Inequities in healthcare and antibiotic access

LMICs grapple with diagnostic shortages and counterfeit drugs, for example, 32% of antibiotics in Cameroon being substandard (Basco, 2004). Women, often caregivers or livestock handlers, face higher MDR exposure due to limited healthcare access, exacerbating gender disparities in treatment outcomes (Iskandar et al., 2020). In Rwanda, 55% of farmers use over-the-counter antibiotics, driven by poverty and lack of veterinary oversight (Manishimwe et al., 2017).

#### Role of international organizations

The main functions expected to be performed by the global health systems include: management of cross-border externalities, mobilizing global support for disadvantaged populations, performing oversight functions on the overall performance of the system and ensuring that adequate global public goods are provided (Moon et al., 2017) (**Table 4**).

**Table 4:** Pivotal roles of international organizations in controlling AMR.

Regulatory bodies	Main Focus	Action plan
World Health Organization (WHO)	To provide global leadership in AMR through initiatives	Implementation of Antimicrobial Stewardship Programs (ASPs) as part of national AMR action plans (World Health Organization (WHO), 2021).
Food and Agriculture Organization (FAO)	Promotion of antibiotics prudent use in agriculture	Improvement in food production systems practices to minimize the reliance on antimicrobials (Food and Agriculture Organization (FAO), 2019).
World Organization for Animal Health (WOAH)	Provision of supports for veterinary ASPs for responsible use of antibiotics in animals,	Promoting of data collection on antimicrobial usage patterns (World Organisation for Animal Health (WOAH), 2021).
National Regulatory Bodies	Regulation of antibiotics sales and distribution	Enforcement of prescription-only policies, and Monitoring the development of resistance trends

The WHO’s Global Antimicrobial Resistance and Use Surveillance System (GLASS) tracks resistance patterns, but only 20% of sub-Saharan African nations fully participate due to resource constraints (WHO, 2023). FAO and WOAH advocate phasing out antibiotic growth promoters, yet enforcement varies, with 50% of LMICs lacking national action plans (FAO, 2024) (**Table 5**).

**Table 5:** GLASS participation by region.

Region	Countries participating	Data completeness (%)	Challenges	Reference
Sub-Saharan Africa	20%	30%	Lab shortages	(WHO, 2023)
South Asia	60%	50%	Underreporting	(Africa CDC, 2023)
Latin America	70%	60%	Funding gaps	(Restrepo-Arbeláez et al., 2023)

Based on WHO and regional reports. * Global Antimicrobial Resistance and Use Surveillance System (GLASS).

Surveillance bottlenecks such as underreporting, limited laboratory capacity (only 25% of African hospitals are equipped for resistance testing), and poor data interoperability hamper global surveillance efforts (WHO, 2023). Nigeria’s National Action Plan for AMR (NAP-AMR) highlights that 50% of its labs lack resistance testing capabilities, delaying timely interventions (Nigeria Centre for Disease Control, 2023a). These global challenges underscore the need for a One Health approach, integrating human, animal, and environmental strategies to combat MDR effectively.

### One health perspective

#### Transmission cycles

MDR bacteria cycle bidirectionally between humans, animals, and environments, with 60% of human pathogens being zoonotic, such as Escherichia coli and Salmonella (Klous et al., 2016). For example, in Ghana, ESBL-producing E. coli shared between poultry and farmers highlights occupational risks, driven by close contact in agricultural settings (Falgenhauer et al., 2019).

#### Operational integration of one health

operates through national task forces, as exemplified by Nigeria’s NAP-AMR, where human, veterinary, and environmental ministries collaborate on surveillance and policy (Nigeria Centre for Disease Control, 2023a). Inter-ministerial platforms facilitate data-sharing, reducing response times and aligning interventions across sectors (Liu et al., 2016).

#### Case study of colistin resistance in China

In 2015, China’s identification of the mcr-1 gene in livestock sparked global concern, as it spread via international trade (Charoenboon et al., 2019). A 2017 ban on colistin as a growth promoter reduced its prevalence by 40%, demonstrating the power of One Health-driven policy interventions (Charoenboon et al., 2019). Operational integration paves the way for comprehensive One Health strategies and this is evidenced by global successes.

**Figure 2:**
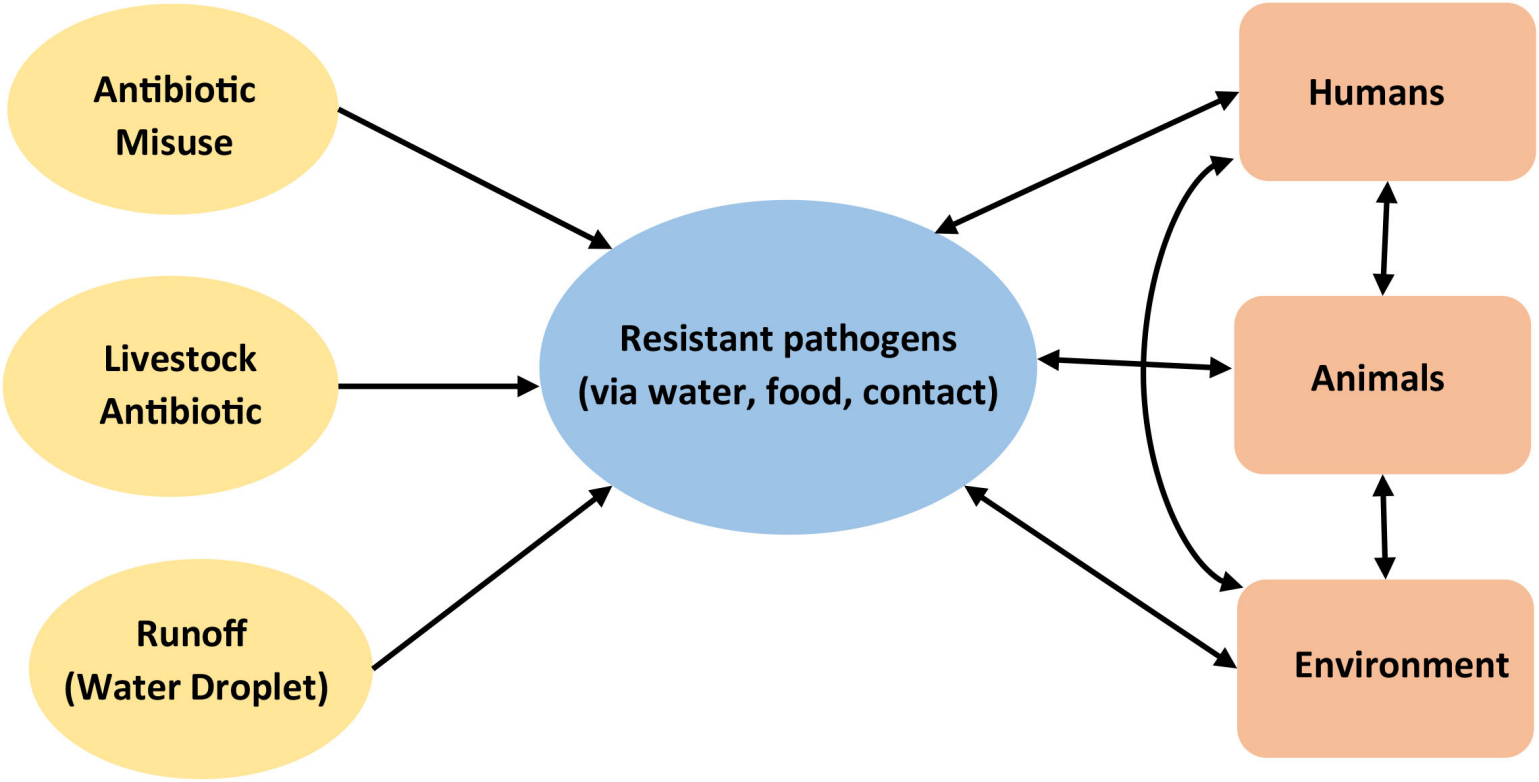
One health AMR transmission routes.

The dynamic relationships within the ecosystem (**Figure 2**) depicts the interactions between humans, animals and the environment and how any misuse of or release of antibiotics (inputs such as antibiotic misuse, livestock antibiotics, runoff (water droplet) disrupts the equilibrium the entire system through the release of deleterious outputs in this case-resistant pathogens via water, food and contact with negative impact.

#### Case studies of successful interventions

**Figure 3:**
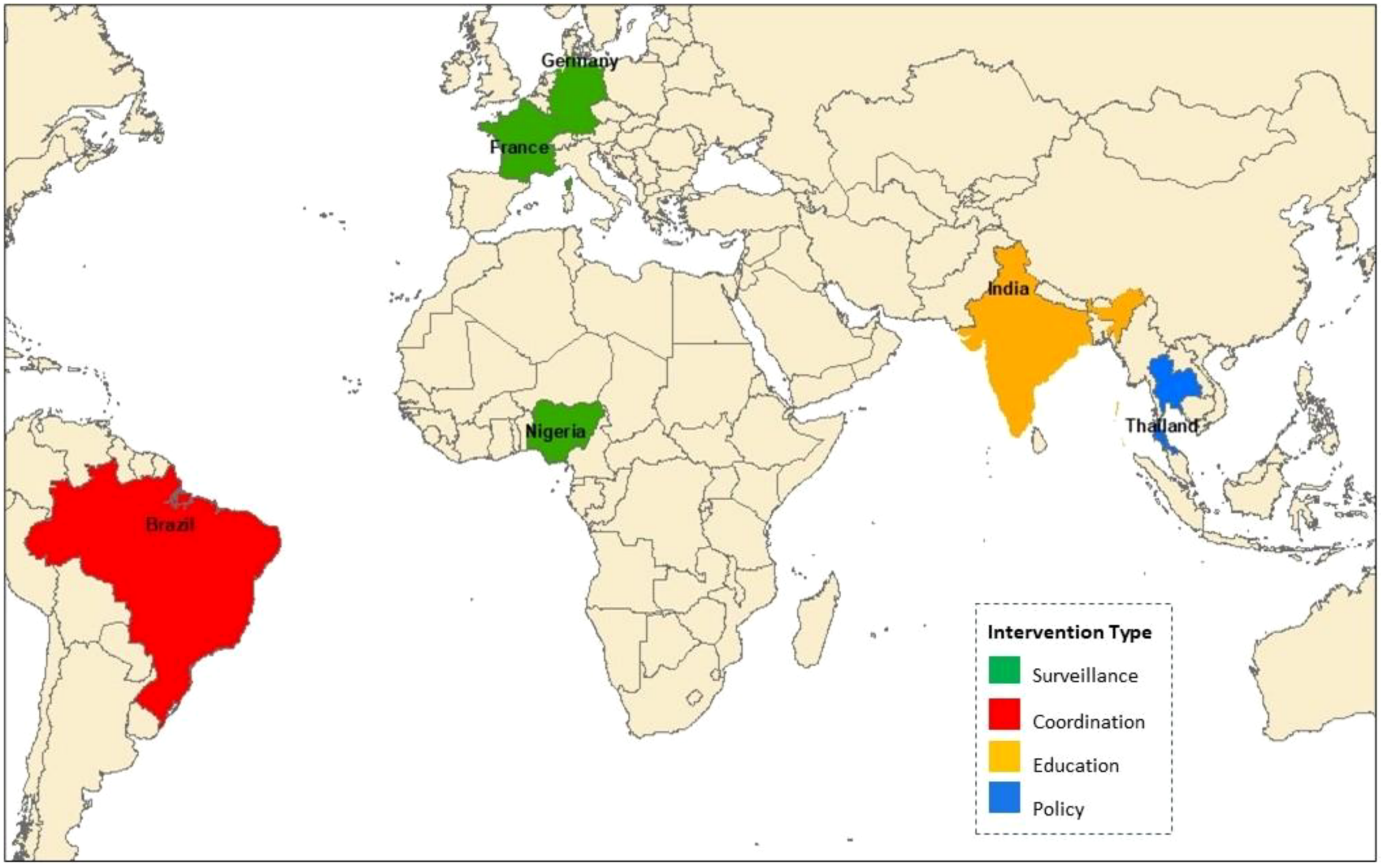
Map showing countries and their primary intervention strategy against MDR infections.

Thailand’s 2017–2021 National Strategic Plan reduced antibiotic use by 30% through regulatory reforms (Charoenboon et al., 2019). The EU’s surveillance network lowered resistance rates by enhancing data-sharing (Authority and EMA, 2021). Brazil’s 2024 AMR plan strengthened intersectoral coordination (Ministério da Saúde (Brazil), 2018). Nigeria’s NAP-AMR improved surveillance coverage (Nigeria Centre for Disease Control, 2023b). And India’s Red Line campaign cut antibiotic misuse by 20% (Ranganathan and Ranjalkar, 2023) (**Figure 3**).

These diverse, well-documented successes (**Table 6**) inform scalable One Health models. Brazil’s intersectoral task forces exemplify effective coordination, sharing data through joint risk assessments (Ranganathan and Ranjalkar, 2023). Ghana’s Antimicrobial Awareness Week, reducing misuse by 15%, demonstrates the power of community-driven initiatives to complement policy efforts (Tsekleves et al., 2023).

**Table 6:** Successful one health interventions.

Country	Focus	Outcome	Reference
Thailand	Policy reform	30% antibiotic reduction	(Charoenboon et al., 2019)
EU	Surveillance	Reduced resistance rates	(Authority and EMA, 2021)
Brazil	Task forces	Enhanced coordination	(Ministério da Saúde (Brazil), 2018)
Nigeria	NAP-AMR	Improved surveillance	(Nigeria Centre for Disease Control, 2023b)
India	Red Line campaign	20% misuse reduction	(Ranganathan and Ranjalkar, 2023)

*Outcomes from Global One Health Initiatives.

### Challenges and limitations

Despite the successes of One-Health interventions, persistent challenges in coordination and research gaps necessitate innovative solutions. Bureaucratic fragmentation often divides human and veterinary health agencies, delaying coordinated responses to MDR outbreaks (Masud et al., 2023; Sanga et al., 2024). In LMICs, only 25% of hospitals have laboratories equipped for resistance testing, severely limiting surveillance capabilities (WHO, 2023). Limited political will and insufficient funding exacerbate these issues, with global surveillance systems like GLASS suffering from underreporting, particularly in sub-Saharan Africa (WHO, 2023). A critical research gap lies in the paucity of longitudinal studies linking environmental contamination to clinical infections, hindering evidence-based policy development (Wang et al., 2022). **Table 7** summarizes these gaps, highlighting priority areas for future investigation to strengthen One Health approaches. Addressing these challenges requires forward-thinking strategies, leveraging technology and community action to drive progress.

**Table 7:** Key research gaps in MDR management.

Domain	Research gap	Priority need	Reference
Clinical	Impact of point-of-care diagnostics in LMICs	Scalability studies	(Tran et al., 2023)
Environmental	Longitudinal environmental-clinical linkages	Cohort studies	(Wang et al., 2022)
Global	Cross-border transmission dynamics	Real-time surveillance	(Arcilla et al., 2017)

*Priorities for future AMR research.

### Integrated one health approach

Collaboration is a participatory process of engaging key actors in addressing complex problems that cannot be handled by a single entity. Intersectoral collaborations creates the needed opportunity to strengthen the health system through increasing coverage, expanding access and improving the comprehensive availability of MDR services across communities.

With respect to MDR infections, an integrated One Health approach implies collaboration across human, animal, and environmental health sectors to address the multifaceted challenges of MDR bacterial infections.

**Figure 4:**
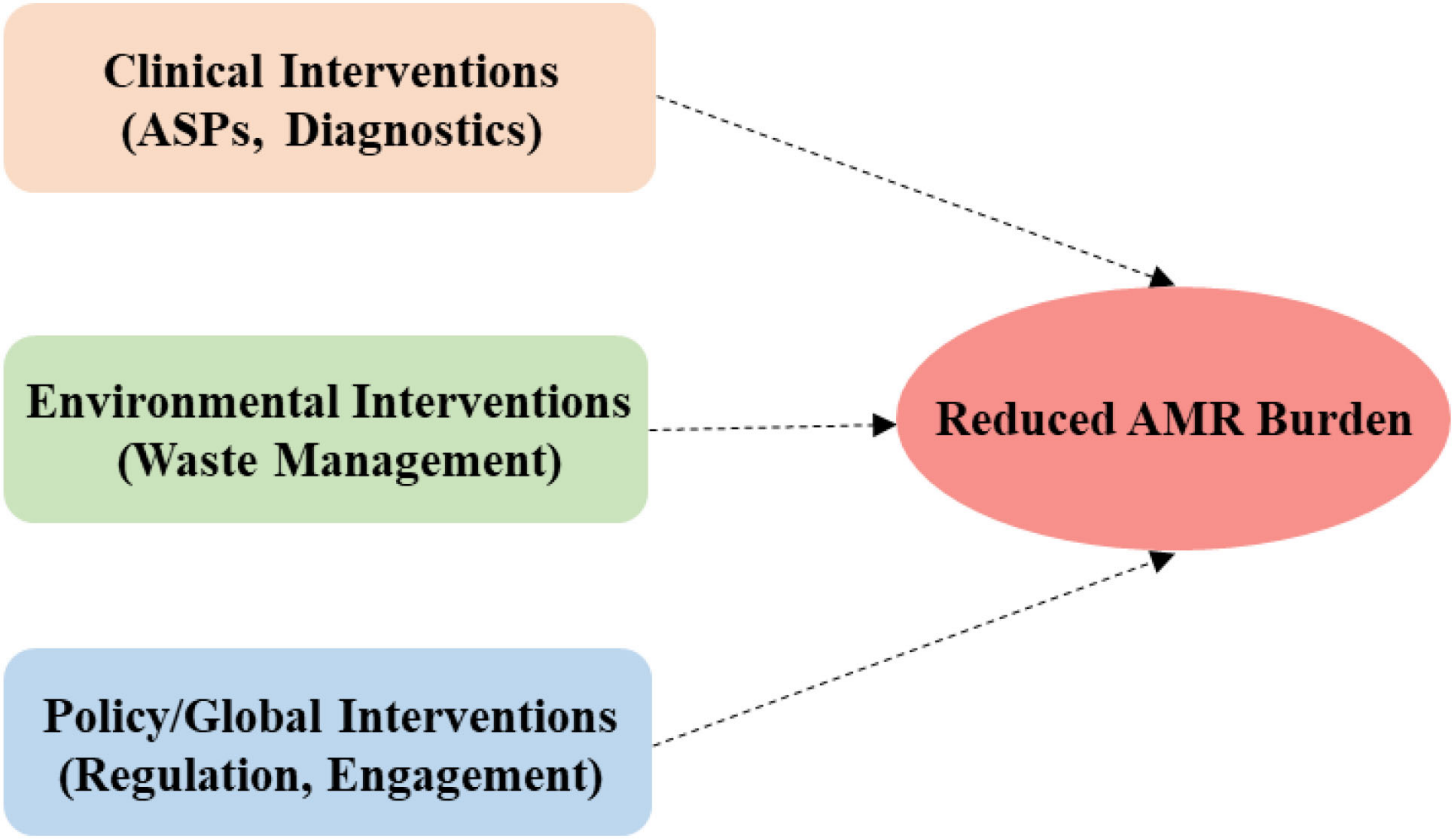
Integrated one health framework for MDR response.

**Figure 4** depicts a One Health Framework based on interconnected interventions, critical for reducing MDR burden holistically across domains. The focus is on intentional linking of clinical (ASPs, diagnostics), environmental (waste management), and policy/global (regulation, engagement) interventions with a clear convergence with reduced AMR burden as the output. This Framework projects that effective partnerships involving veterinarians, environmental health scientists, public health officials, and clinicians working together to develop targeted interventions that address resistance at its sources.

### Future directions and recommendations

#### AI-driven solutions

The application of AI to manage AMR is fast gaining ground (Mansur et al., 2023). The potential for integrating technology (e.g., AI, Machine Learning (ML)) for real-time MDR monitoring and tailored interventions can be achieved through various ways to enhance effective healthcare delivery. The integration of AI and ML in diagnostics and treatment planning revolutionizing patient care, particularly ML, in medical diagnostics is ushering in a new era of healthcare. This revolutionary approach is characterized by increased accuracy, efficiency, and speed, surpassing traditional human capabilities in certain aspects.

Giving practical examples, in Ethiopia’s rural clinics, a young mother’s life was saved when WHO’s 2024 AMASS tool identified a resistant *Escherichia coli* infection in hours, cutting diagnostic time by 50% and guiding precise therapy (Martin, 2021). AI’s Machine Learning capabilities, as demonstrated by IDseq’s global deployment, analyze genomic and clinical data to predict resistance patterns with 90% accuracy, revolutionizing MDR management (Luca, 2024).

However, challenges persist: algorithm bias from unrepresentative datasets, non-standardized data inputs, and infrastructure gaps in LMICs—where only 10% of laboratories have cloud connectivity—limit scalability (Tran et al., 2023). Ethical concerns, such as patient data privacy and equitable access, further complicate adoption, necessitating robust governance frameworks (Schar et al., 2021) Despite these hurdles, AI’s transformative potential, when paired with investments in LMIC infrastructure, offers a pathway to precision interventions and global health equity.

#### Community engagement

Community-driven initiatives, such as Ghana’s Antimicrobial Awareness Week (15% misuse reduction) and India’s Red Line campaign (20% reduction), demonstrate the power of public education (Tsekleves et al., 2023; Sanga et al., 2024). Women, often primary caregivers and livestock handlers, face heightened MDR exposure due to limited healthcare access, requiring targeted education to address gender disparities and reduce transmission risks (Iskandar et al., 2020).

#### Policy reforms

Global bans on antibiotic growth promoters, public-private funding models, and standardized surveillance systems, as implemented in Brazil’s 2024 AMR plan, are essential for sustainable progress (Ranganathan and Ranjalkar, 2023). Nigeria’s NAP-AMR, emphasizing laboratory upgrades, provides a replicable model for LMICs to strengthen diagnostic capacity (Ministério da Saúde (Brazil), 2018). Clinical misuse, environmental reservoirs, and global spread, particularly in LMICs, thrive in the absence of coordinated responses. Thailand’s 30% reduction in antibiotic use through policy reform and AI’s 50% improvement in diagnostic speed offer beacons of hope, yet gender inequities and surveillance gaps persist (Authority and EMA, 2021; Martin, 2021).

**Figure 5:**
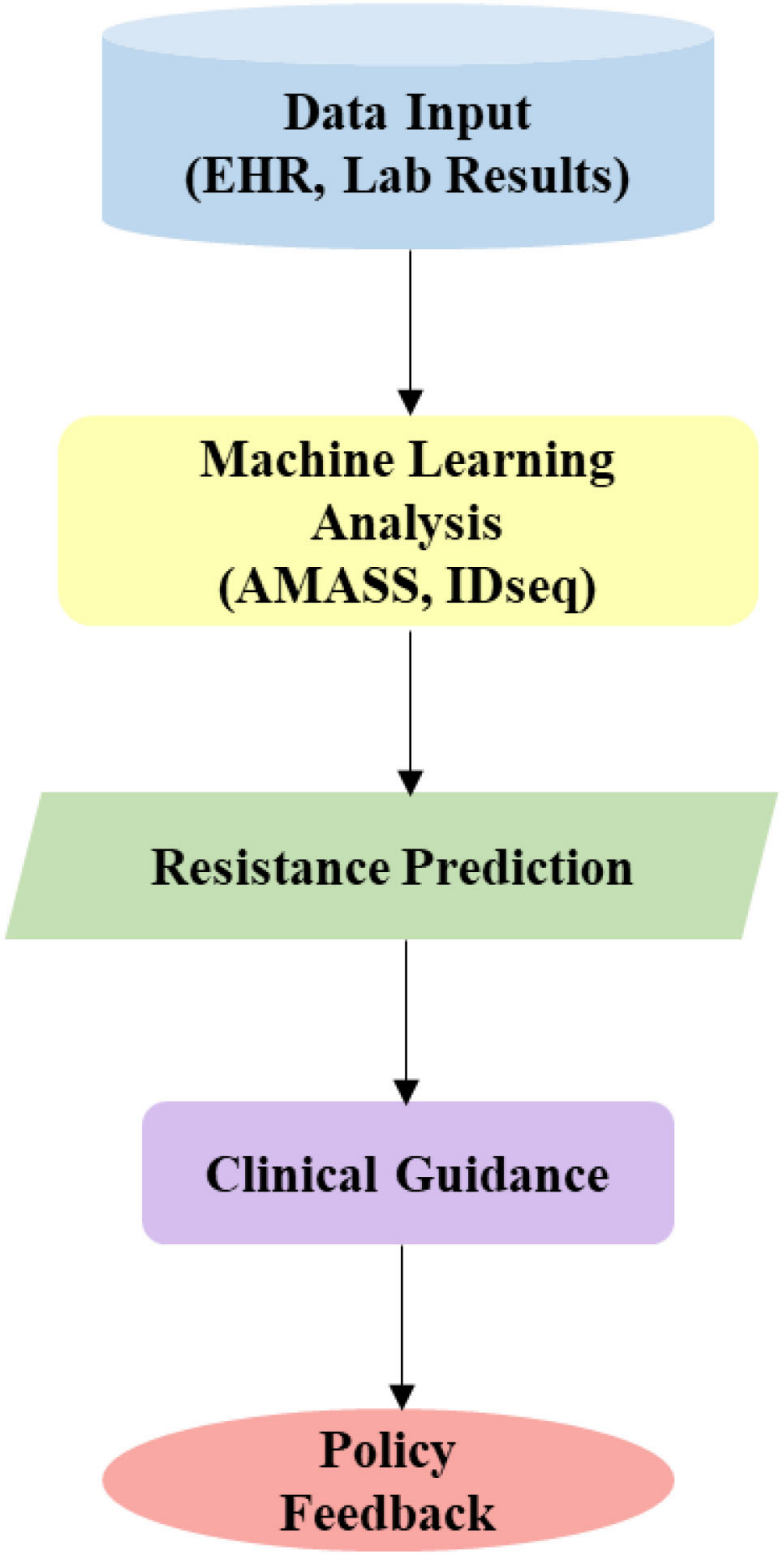
AI workflow for MDR surveillance.

We present a flowchart of an AI workflow from data input (EHR, lab results) to ML analysis (AMASS, IDseq), resistance prediction, clinical guidance, and policy feedback (**Figure 5**). This will enable rapid MDR detection, critical for precision interventions, prompt community engagement and policy development.

These strategies converge in a bold vision for global health security, as outlined in our concluding call to action.

## Conclusion

This review has given deep insight into the problems associated with antimicrobial resistance and the various drivers of MDR bacterial infections. We reiterated that the One Health approach is important for combating MDR bacterial infections because it recognizes the interconnectedness of human, animal, and environmental health. With respect to MDR infections, an integrated One Health approach implies collaboration across human, animal, and environmental health sectors to address the multifaceted challenges of MDR bacterial infections. In tackling the identified gaps, proactively incorporating AI technology for real-time drug resistance monitoring will go a long way to help in combating the problems of MDR infections in communities. Although the use of antibiotics for bacterial infection control is still very important, considering reduction in its use in all facets of One Health and focusing on alternative strategies to limit the proliferation of drug-resistant pathogens is highly recommended. Since antibiotic resistance is as a result of abuse, antibiotic-free technique is an alternative approach to dealing with the present antibacterial challenges.

In summary, a One Health approach that integrates human, animal, and environmental based strategies, is not merely an option but an imperative. A proactive paradigm shift focusing on transformative actions to:

- Fund intersectoral surveillance: Expand the WHO GLASS participation and laboratory capacity to track resistance in real time.
- Scale AI equitably: Invest in LMICs infrastructure to ensure inclusive, ethical technology deployment.
- Empower communities: Prioritize gender-focused education to curb antibiotic misuse and reduce exposure.

These steps, grounded in One Health principles, are critical to preserving antibiotics and securing a resilient global health future.
